# Replication Characteristics of African Swine Fever Virus (ASFV) Genotype I E70 and ASFV Genotype II Belgium 2018/1 in Perivenous Macrophages Using Established Vein Explant Model

**DOI:** 10.3390/v16101602

**Published:** 2024-10-12

**Authors:** Shaojie Han, Dayoung Oh, Nadège Balmelle, Ann Brigitte Cay, Xiaolei Ren, Brecht Droesbeke, Marylène Tignon, Hans Nauwynck

**Affiliations:** 1 Laboratory of Virology, Department of Translational Physiology, Infectiology and Public Health, Faculty of Veterinary Medicine, Ghent University, Salisburylaan 133, 9820 Merelbeke, Belgium; 2 Service Viral Re-Emerging, Enzootic and Bee Diseases, Department Infectious Diseases in Animals, Sciensano, Groeselenbergstraat 99, 1180 Brussels, Belgium

**Keywords:** ASFV, vein explant, perivenous macrophage, CD163

## Abstract

African Swine Fever Virus (ASFV), resulting in strain-dependent vascular pathology, leading to hemorrhagic fever, is an important pathogen in swine. The pathogenesis of ASFV is determined by the array and spatial distribution of susceptible cells within the host. In this study, the replication characteristics of ASFV genotype I E70 (G1-E70) and ASFV genotype II Belgium 2018/1 (G2-B18) in the environment of small veins were investigated in an established vein explant model. Immunofluorescence staining analysis revealed that perivenous macrophages (CD163^+^ cells) were widely distributed in the explant, with most of them (approximately 2–10 cells/0.03 mm^2^) being present close to the vein (within a radius of 0–348 µm). Upon inoculation with G1-E70 and G2-B18, we observed an increase in the quantity of cells testing positive for viral antigens over time. G1-E70 replicated more efficiently than G2-B18 in the vein explants (7.6-fold for the ear explant at 72 hpi). The majority of ASFV^+^ cells were CD163^+^, indicating that macrophages are the primary target cells. Additional identification of cells infected with ASFV revealed the presence of vimentin^+^, CD14^+^, and VWF^+^ cells, demonstrating the cellular diversity and complexity associated with ASFV infection. By the use of this new vein explant model, the susceptibility of vascular and perivascular cells to an ASFV infection was identified. With this model, it will be possible now to conduct more functional analyses to get better insights into the pathogenesis of ASFV-induced hemorrhages.

## 1. Introduction

African Swine Fever (ASF) has a serious impact on the global pork industry, with profound effects on animal welfare and the economy [1]. It jeopardizes a stable and sustainable supply of pork, which is important for global food security. The etiological agent of ASF is an enveloped, double-stranded DNA virus known as ASFV, with a diameter of approximately 250 nm [2]. Its genome contains more than 150 ORFs, which encode proteins with multiple functions during replication in susceptible cells of ticks and pigs (review in [3]). P72 has been extensively researched for the advancement of virological and serological diagnostic techniques, molecular genotyping of ASFV strains, and the production of specific antibodies against ASFV [4,5,6,7].

ASFV genotype II emerged for the first time in Belgium in September 2018 [8,9] and spread in wild boar until complete eradication one year later [10]. Upon experimental inoculation, the typical pathomorphological and histopathological lesions were reproduced in an acute and severe disease model, demonstrating the fidelity of the experimental system [11]. ASFV is characterized by virulence-dependent hemorrhagic fever in swine, leading to vascular pathology [12]. Infected pigs frequently show discoloration patterns at various parts of the body, such as the ears, distal extremities, and abdomen, and severe hemorrhages can be observed internally in different organs, such as lungs, lymph nodes, spleen, and kidneys [11,13]. This phenomenon is associated with thrombocytopenia, augmented vascular permeability, and extravasation of blood components [14]. The secretion of proinflammatory cytokines by pulmonary intravascular macrophages has been associated with vascular damage [12,15].

The pathogenesis of ASFV is determined by the array and spatial distribution of susceptible cells within the host. Similarly to the porcine reproductive and respiratory syndrome disease virus (PRRSV) [16], macrophages are the primary cellular targets for ASFV [12,13]. Due to their central role in the immune response to pathogens, macrophages are primarily affected by ASFV infections, leading to impaired immunity [17]. The number of macrophages in different organs increases during ASFV replication in vivo [12]. The restricted cellular tropism suggests the use of a restricted number of receptors for ASFV infection [18]. The scavenger receptor CD163, a surface marker of macrophages, is widely recognized as an essential mediator for PRRSV infection [19,20]. A previous study has demonstrated the involvement of CD163 in ASFV infection [21]. Nevertheless, in another study, it was observed that CD163-knockout pigs were still susceptible to ASFV, demonstrating that CD163 is not the only entry mediator for ASFV infection [22]. Vimentin is prominently expressed in monocytes and some macrophages [23], and interacts with pD1133L of ASFV (an intermediate-late protein), promoting virus replication [24,25]. Additionally, it has been reported that ASFV is capable of replicating in various cell types, such as hepatocytes, nasal epithelial cells, and endothelial cells [12,15,26,27,28]. Hence, this implies that more than one cellular receptor is used by ASFV.

The advance of a potent vaccine is essential to prevent ASFV infections and transmission in domestic pigs and wild boar. Vaccine development upon natural attenuation or genetic engineering (MLV) is ongoing [29,30,31]. During the first use of commercially licensed vaccines in Vietnam in 2023, pig mortalities after the first vaccination campaign raised controversial opinions about the efficacy and safety of the MLV vaccine(s), particularly in cases of improper use [32]. Currently, the scientific community is advocating for standardized guidelines and criteria regarding the purity, potency, and safety of ASF MLV vaccines [33]. A better understanding of the interaction of viruses and susceptible cells will be of great use for the design of new vaccines and disease-resistant gene-edited pigs.

Recently, a new vein explant model has been developed for the study of PRRSV in the perivenous environment [34]. This study demonstrated that the highly pathogenic type 1 PRRSV strain Lena exhibited more efficient replication in perivenous macrophages located within vein explants compared to the low pathogenic strain (LV). The involvement of perivenous macrophages in the pathogenesis of ASFV infection remains inadequately elucidated. Consequently, in the present work, we aim to evaluate specifically the susceptibility of perivenous macrophages to ASFV and further characterize ASFV-susceptible cells in the vein explant model to gain a more comprehensive understanding of the pathogenesis of vein pathology induced by ASFV.

## 2. Materials and Methodology

### 2.1. Animals and Collection of Tissues

Three-week-old conventional pigs, originating from an ASFV and PRRSV-negative farm, were used for tissue collection. The tissues were collected and prepared following previously established protocols [34]. To evaluate the viability of the explants, an in situ Cell Death Detection Kit (Roche Diagnostics Corporation, Basel, Switerland) was employed as described in a previous report [34].

### 2.2. Virus

Two ASFV strains were used in this study: a third passage of genotype I strain E70 (Spanish isolate obtained in 1970, further referred to in this study as G1-E70) in porcine alveolar macrophages (PAMs) (genotype I, 10^6.38^ TCID_50_/mL) and a sixth passage of Belgium 2018/1 (Belgian isolate obtained in 2018 from a dead ASFV-positive wild boar, Etalle, Belgium) [8] in PAMs (genotype II, 10^7.47^ TCID_50_/mL), further referred to in this study as G2-B18. The viral titers were assessed in porcine alveolar macrophages and quantified using the Reed and Muench method [35]. Given the variations of the cells used for virus production and titration, the titration results were confirmed by PCR. The Belgium 2018/1 was diluted to the same titer (10^6.38^ TCID_50_/mL) as E70 before inoculation in a complete RPMI medium [34].

### 2.3. Inoculation of the Explants

After incubating for 1 h at 37 °C in the presence of 5% CO_2_, the explants were inoculated with 500 μL of each virus suspension per well (containing 10^6.08^ TCID_50_ virus). An additional explant was inoculated with 500 μL of the complete medium as a mock inoculum. Following a one hour inoculation, another 500 μL of complete medium was added to each well. Samples were collected at 0, 24, 48, and 72 h post-inoculation (hpi), followed by three washes and embedding in methylcellulose medium (Thermo Fisher GmbH, Kandel, Germany) before being frozen at −70 °C.

### 2.4. Analysis of Perivenous Macrophages Using Immunofluorescence (IF) Staining

The collected frozen samples were processed following previously established protocols before IF staining [34]. In order to quantify and identify the susceptible cells in our model, we conducted double immunofluorescence staining targeting CD163 and ASFV VP72. Sections were incubated with a mouse monoclonal antibody (mAb) against porcine CD163 (clone 2A10/11, IgG1, Bio-Rad, Oxford, UK, 1:200), and mouse FITC-conjugated anti-ASFV VP72 monoclonal antibody (lgG2a, 18BG3, 1:100, FITC-conjugated, Ingenasa, Madrid, Spain) for 1 h at 37 °C [4], followed by PBS washing and incubation with goat anti-mouse IgG1 Alexa fluor 594 (1:200, Invitrogen, Waltham, MA, USA) for 1 h at 37 °C. Mouse anti-PRV mAb clone 13D12 (IgG1) was introduced as isotype control [36]. Non-specific binding sites were blocked with normal goat serum (10%), and cell nuclei were subsequently counterstained with Hoechst33342 (10 μg/mL, Invitrogen) for ten minutes at 37 °C.

The replication kinetics of ASFV in vein explants were assessed by quantifying the number of ASFV-infected cells throughout the entire cryosection at different time points after inoculation using a Nikon Eclipse Ts2R-FL inverted microscope (Nikon, Melville, NY, USA). Additionally, the total number of CD163^+^ cells and the number of ASFV-infected CD163^+^ cells (CD163^+^ASFV^+^) were quantified at different regions of interest (ROIs) (10× ocular lens and 40× objective) adjacent to each other in the depth of the explants (Region A (0–174 µm), Region B (174 µm–348 µm), Region C (348 µm–522 µm), and Region D (522 µm–696 µm)), as previously documented [34]. The area of each region was 0.03 mm^2^.

### 2.5. Further Identification of ASFV Susceptible Cells by IF Staining

Antibodies against different cell surface markers (Table 1) were used to further identify the viral antigen-positive cells by IF staining. To avoid cross-reaction between antibodies, two different primary antibodies against ASFV VP72 (18BG3, IgG2a, and 1BC11, IgG1) together with different cell marker antibodies were used (Table 1). The sections were first incubated with the primary antibodies for 1 h at 37 °C, followed by a washing step with PBS, and further incubated with a combination of isotype and host species-matched secondary antibodies for 1 h at 37 °C: goat anti-mouse IgG1 Alexa fluor 594 (1:400, Invitrogen, Waltham, MA, USA), goat anti-mouse IgG1 FITC (1:500, Invitrogen), goat anti-mouse IgG2a Alexa fluor 594 (1:500, Invitrogen), goat anti-mouse IgG2b Alexa fluor 594 (1:200, Invitrogen), or goat anti-rabbit IgG Alexa fluor 594 (1:200, Invitrogen).

Cell nuclei were counterstained with Hoechst 33342 (10 μg/mL, Invitrogen) for 10 min at 37 °C. Cell marker-positive cells were analyzed within the population of ASFV^+^ cells and the results were shown as percentages (cell surface marker^+^ ASFV^+^/total ASFV^+^ × 100). All ASFV^+^ cells were quantitated within the whole cryosection and further characterized based on the expression of the cell markers.

### 2.6. Statistical Analysis

All statistical analyses were performed using GraphPad Prism version 9.0 (GraphPad, San Diego, CA, USA). Differences between sample groups were assessed using multiple-way analysis of variance (ANOVA), followed by Tukey’s comparison post hoc test. The data are presented as mean ± standard deviation (SD) from three independent experiments. Results with a *p*-value of <0.05 were considered statistically significant.

## 3. Results

### 3.1. Evaluation of Explant Viability of Ear and Leg Vein Explants

**Figure 1:**
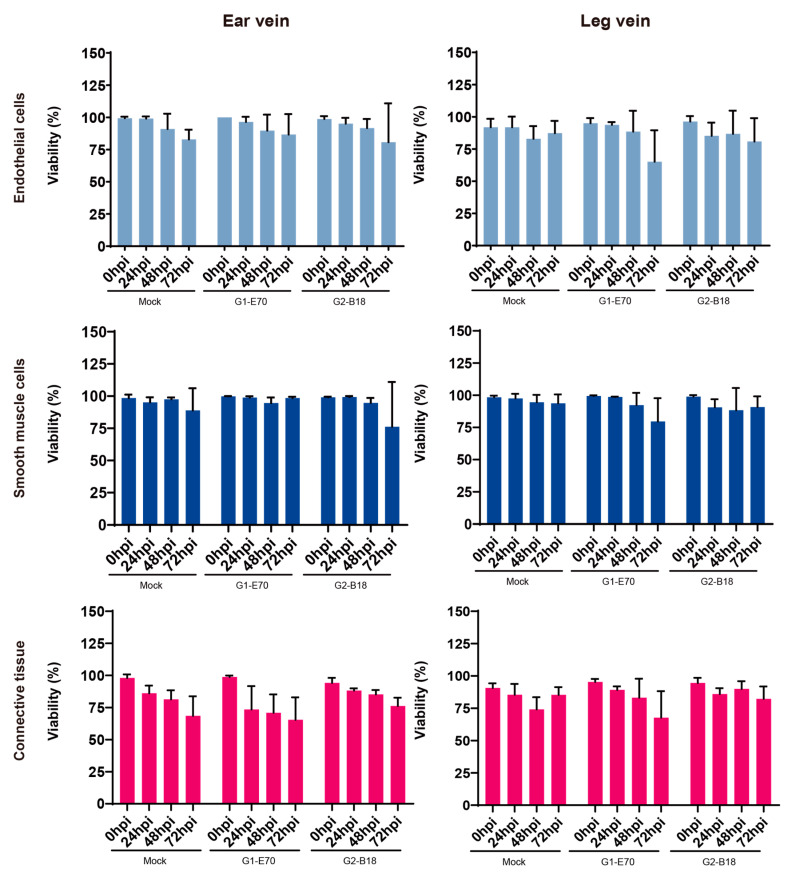
Evaluation of the effect of in vitro culture and ASFV infection on the viability of ear and leg vein explants. The number of TUNEL-positive cells within the different parts of explants was quantified after selecting three random areas. All data are expressed as the mean value of three experiments ± SD.

As shown in Figure 1, in order to assess the impact of in vitro growth and ASFV infection on the survival of vein explants, the proportion of TUNEL-positive cells was determined after 0, 24, 48, and 72 h of cultivation. Although no statistical significance was detected, the viability of explants in the different groups (mock, G1-E70, and G2-B18 inoculated explants) exhibited a trend of decreasing over time. In the ear vein explant, the cell viability of endothelial cells, smooth muscle cells, and connective tissue was reduced at 72 hpi to (i) 82.84 ± 7.64%, 88.84 ± 17.20%, and 68.53 ± 15.22%, respectively, in the mock-inoculated explants, (ii) 86.67 ± 15.88%, 98.46 ± 0.99%, and 65.42 ± 17.56%, respectively, in the G1-E70-inoculated explants, and (iii) 80.67 ± 30.14%, 76.16 ± 34.78%, and 76.20 ± 6.43%, respectively, in the G2-B18-inoculated explants. In the leg vein explant, the cell viability of endothelial cells, smooth muscle cells, and connective tissue was reduced at 72 hpi to (i) 87.34 ± 9.54%, 93.75 ± 6.85%, and 85.37 ± 5.98%, respectively, in mock-inoculated explants; (ii) 65.2 ± 24.36%, 79.57 ± 18.07%, and 67.73 ± 20.47%, respectively, in the G1-E70-inoculated explants; and (iii) 80.82 ± 18.12%, 90.8 ± 8.31%, and 82.29 ± 9.55%, respectively, in the G2-B18-inoculated explants.

### 3.2. Quantitation of Perivenous Macrophages in Ear and Leg Vein Explants

**Figure 2:**
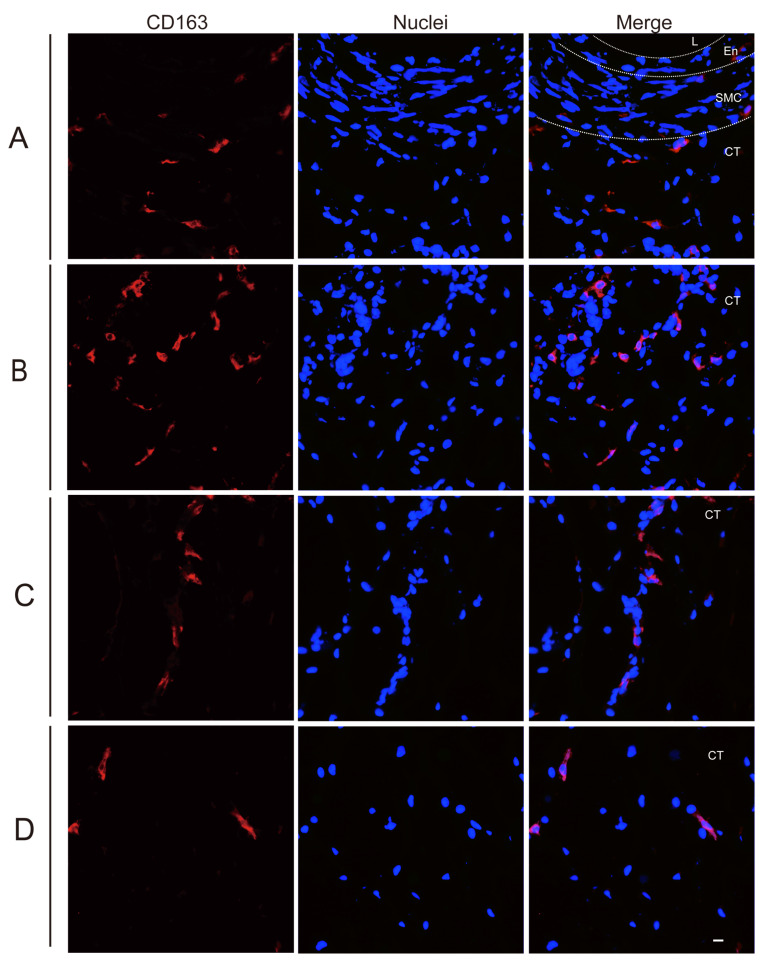
Identification of CD163^+^ cells in ear vein explants by IF staining. Region (**A**) (0–174 µm), Region (**B**) (174–348 µm), Region (**C**) (348–522 µm), and Region (**D**) (522–696 µm) are defined as consecutive layers extending from the endothelial cell layer of the vessel into the connective tissue (0–696 µm). Sections of the ear vein explant were stained with immunofluorescence staining to identify the expression of CD163 (red) after inoculation with G1-E70 or G2-B18, Blue represents cell nucleus staining. L: Lumen, En: Endothelial cell layer, SMC: Smooth muscle cell layer, CT: Connective tissue. Scale bar: 10 μm.

**Figure 3:**
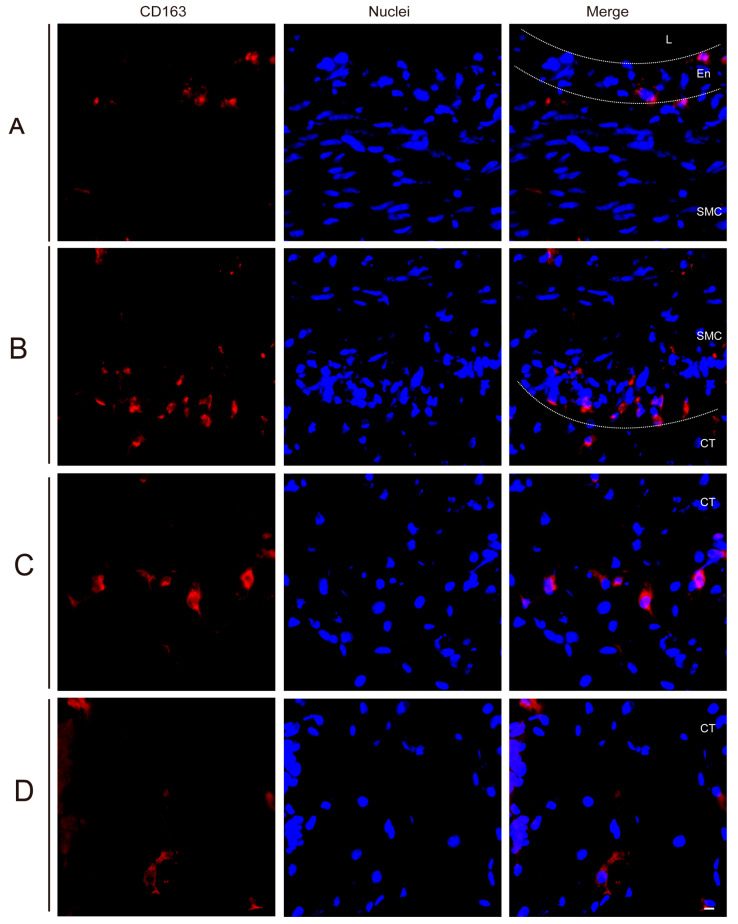
Identification of CD163^+^ cells in the leg vein explants by IF staining. Region (**A**) (0–174 µm), Region (**B**) (174–348 µm), Region (**C**) (348–522 µm), and Region (**D**) (522–696 µm) are defined as consecutive layers extending from the endothelial cell layer of the vessel into the connective tissue (0–696 µm). Sections of the leg vein explant were stained with immunofluorescence staining to identify the expression of CD163 (red) after inoculation with G1-E70 or G2-B18. Blue represents cell nucleus staining. L: Lumen, En: Endothelial cell layer, SMC: Smooth muscle cell layer, CT: Connective tissue. Scale bar: 10 μm.

**Figure 4:**
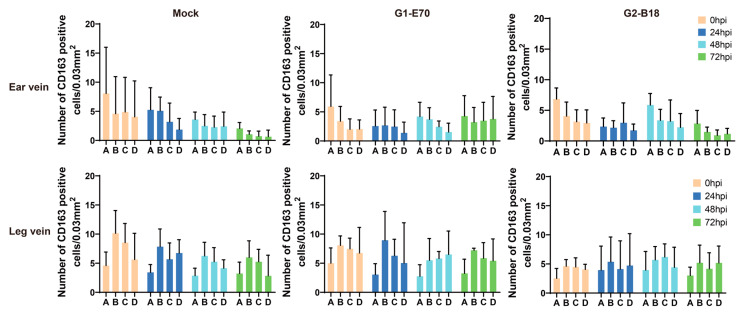
Quantification and distribution of CD163^+^ cells in the ear and leg vein explants. Quantification of perivenous CD163^+^ macrophages in region (**A**) (0–174 µm), region (**B**) (174–348 µm), region (**C**) (348–522 µm), and region (**D**) (522–696 µm) at various time points. All data are expressed as a mean value of three experiments ± SD.

The CD163^+^ cells, also known as perivenous macrophages, were found to be widely spread throughout various areas of ear vein explants and leg vein explants through immunofluorescence stainings (see Figure 2 and Figure 3). To localize CD163^+^ cells in vein explants, four ROIs were identified: region A (0–174 µm), region B (174–348 µm), region C (348–522 µm), and region D (522–696 µm), as previously reported [34]. During the 72 h cultivation period, CD163^+^ cells were found to be distributed extensively throughout various areas of the vein explant (see Figure 4). The majority of CD163^+^ cells were observed in region A (0–174 µm) of the ear vein explant (2.3–8 cells/0.03 mm^2^) and region B (174–348 µm) of the leg vein explant (4.6–10 cells/0.03 mm^2^), showing a decrease as the distance from the vein increased. Few CD163^+^ cells were present in the endothelial cell layer and the smooth muscle cell layer. Overall, the quantity of CD163^+^ cells showed a decreasing pattern with increasing distance from the vein lumen, and this pattern was further intensified with longer incubation times.

### 3.3. Quantitation and Identification of ASFV-Infected Cells in Ear and Leg Vein Explants

**Figure 5:**
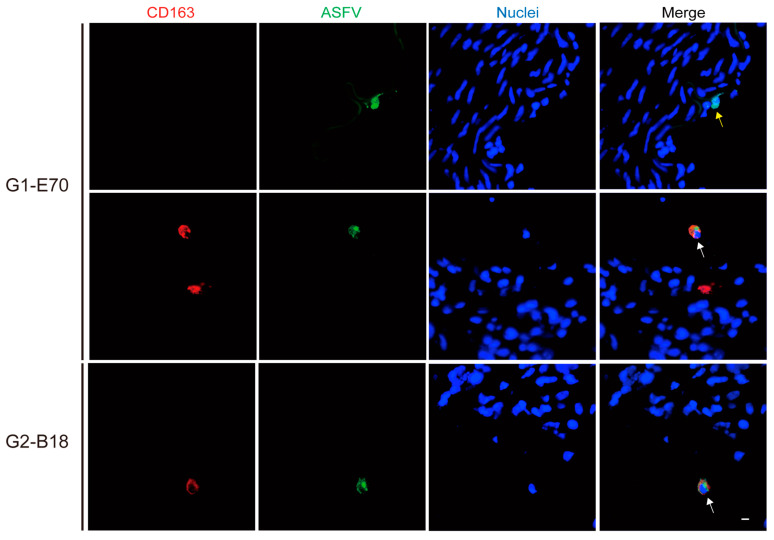
Identification of ASFV-infected cells as CD163^+^ASFV^+^ cells (white arrow) or CD163^−^ASFV^+^ (yellow arrow) cells in ear vein explants. Sections of the ear vein explant were stained with a double IF stain to identify the expression of CD163 (red) and ASFV (green) after being inoculated with G1-E70 or G2-B18. Blue represents cell nucleus staining. ASFV^+^CD163^+^ cells and ASFV^+^CD163^−^ cells are indicated by white arrows and yellow arrows, respectively. Scale bar: 10 μm.

**Figure 6:**
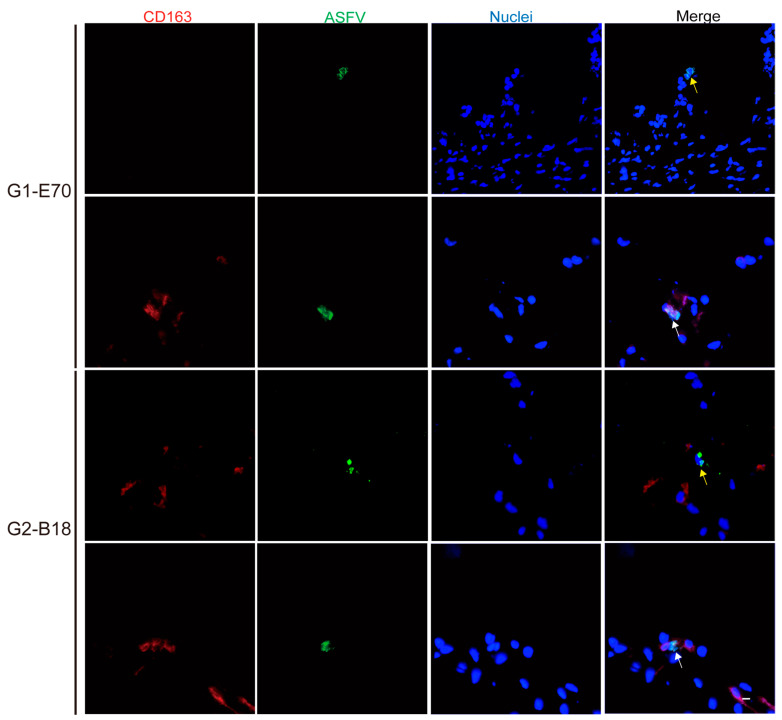
Identification of ASFV-infected cells as CD163^+^ASFV^+^ cells (white arrow) or CD163^−^ASFV^+^ (yellow arrow) cells in leg vein explants. Sections of the ear vein explant were stained with a double IF stain to identify the expression of CD163 (red) and ASFV (green) after being inoculated with G1-E70 or G2-B18. Blue represents cell nucleus staining. ASFV^+^CD163^+^ cells and ASFV^+^CD163^−^ cells are indicated by white arrows and yellow arrows, respectively. Scale bar: 10 μm.

**Figure 7:**
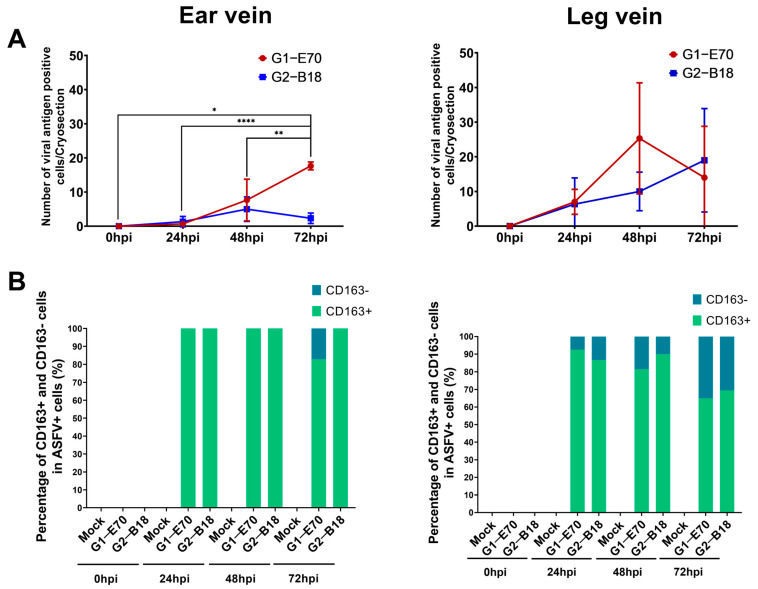
The replication characteristics of ASFV in vein explants were observed at various time points post-inoculation (0, 24, 48, and 72 hpi). (**A**) ASFV-infected cells (G1-E70 and G2-B18) were quantified in whole cryosections of ear and leg vein explants at different time points. (**B**) The proportion of CD163^+^ and CD163^−^ cells in ASFV (G1-E70 or G2-B18) infected cells was measured at various time points in ear vein explants and leg vein explants. Statistical significance was assessed using one-way ANOVA and then followed by Tukey’s multiple comparison post hoc test. (* *p* < 0.05; ** *p* < 0.01; **** *p* < 0.0001). All data are expressed as the mean value of three experiments ± SD.

The quantity and identity of ASFV-infected cells were determined by double IF staining against CD163 and ASFV (VP72) (Figure S1, Figure 5 and Figure 6). The number of ASFV-infected cells enhanced over incubation time (Figure 7).

In the ear vein explants, the number of ASFV-infected cells increased significantly from 1 cell/cryosection at 24 hpi to 17.67 ± 1.15 cells/cryosection at 72 hpi (*p* < 0.0001) in the G1-E70 inoculated group. At 72 hpi, 82.79% of ASFV-infected cells in the G1-E70 group were identified as CD163^+^ (17.21% CD163^−^). In the G2-18 inoculated group, the number of ASFV-infected cells enhanced from 1.30 ± 1.53 cells/cryosection at 24 hpi to a maximum of 5.00 ± 3.61 cells at 48 hpi. All ASFV-infected cells were CD163^+^ in the G2-B18 inoculated group until 72 hpi.

In the leg vein explant, the number of ASFV-infected cells increased from 7.00 ± 3.61 cells/cryosection at 24 hpi to a maximum of 25.33 ± 16.04 cells/cryosection at 48 hpi in the G1-E70 inoculated group. Although the percentage of CD163^−^ cells among all virus-infected cells showed an increasing trend from 7.50% at 24 h to 18.45% at 48 h and 35.00% at 72 h, the majority of ASFV-infected cells were still CD163^+^. In the G2-B18 inoculated group, the number of ASFV-infected cells enhanced from 6.33 ± 7.57 cells/cryosection at 24 hpi to 10 ± 5.57 cells/cryosection at 48 hpi and reached a maximum of 19 ± 14.93 cells/cryosection at 72 hpi. Similarly, most infected cells were CD163^+^ over time.

In summary, G1-E70 replicated more efficiently than G2-B18 in vein explants (the number of G1-E70 infected cells was 7.6 times that of G2-B18 infected cells in ear vein explants at 72 hpi). Most of the ASFV-infected cells were CD163^+^.

### 3.4. Characterization of ASFV Susceptible Cells with Other Cell Markers in Vein Explants

**Figure 8:**
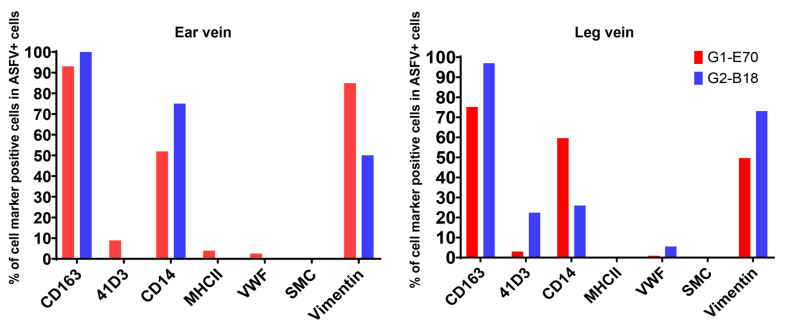
Identification of ASFV-infected cells with different cell markers in vein explants. Viral antigen-positive cells (G1-E70 and G2-B18) were quantitated in ear vein explants and leg vein explants at 72 hpi. Double IF staining was employed to further identify the ASFV-susceptible cells in the ear vein and leg vein explants, using different cell markers. All data are expressed as a mean value of three experiments ± SD.

Not only CD163^+^ cells were susceptible to ASFV. Additionally, some CD163^−^ cells were observed within viral ASFV-infected cells in vein explants (Figure 5 and Figure 6). To further characterize the ASFV-susceptible cells, antibodies against different cell surface markers were used (Figure 8 and Table S1).

**Figure 9:**
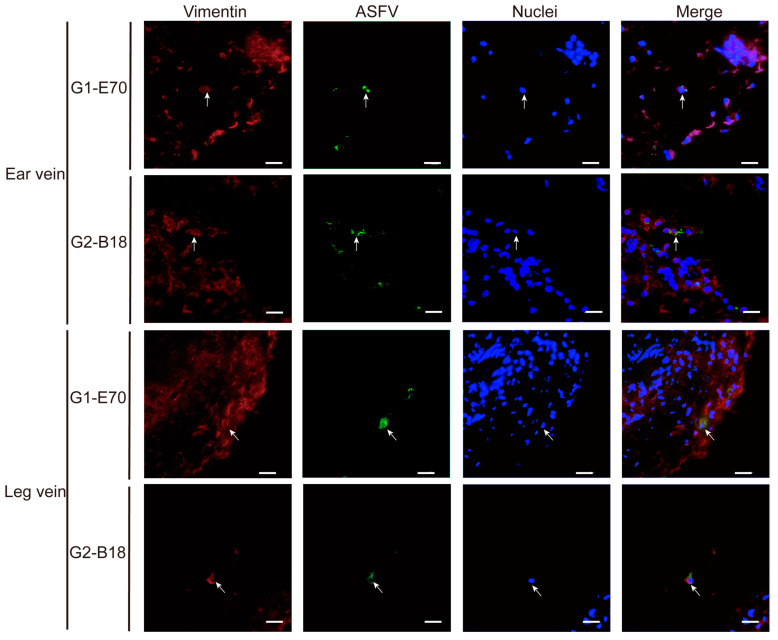
Expression of vimentin in ASFV-infected cells in ear and leg vein explants. Sections of the vein explants were stained with a double IF stain to identify the expression of vimentin (red) and ASFV (green) after being inoculated with G1-E70 or G2-B18. Blue represents cell nucleus staining. ASFV^+^Vimentin^+^ cells are indicated by white arrows. Scale bar: 50 μm.

**Figure 10:**
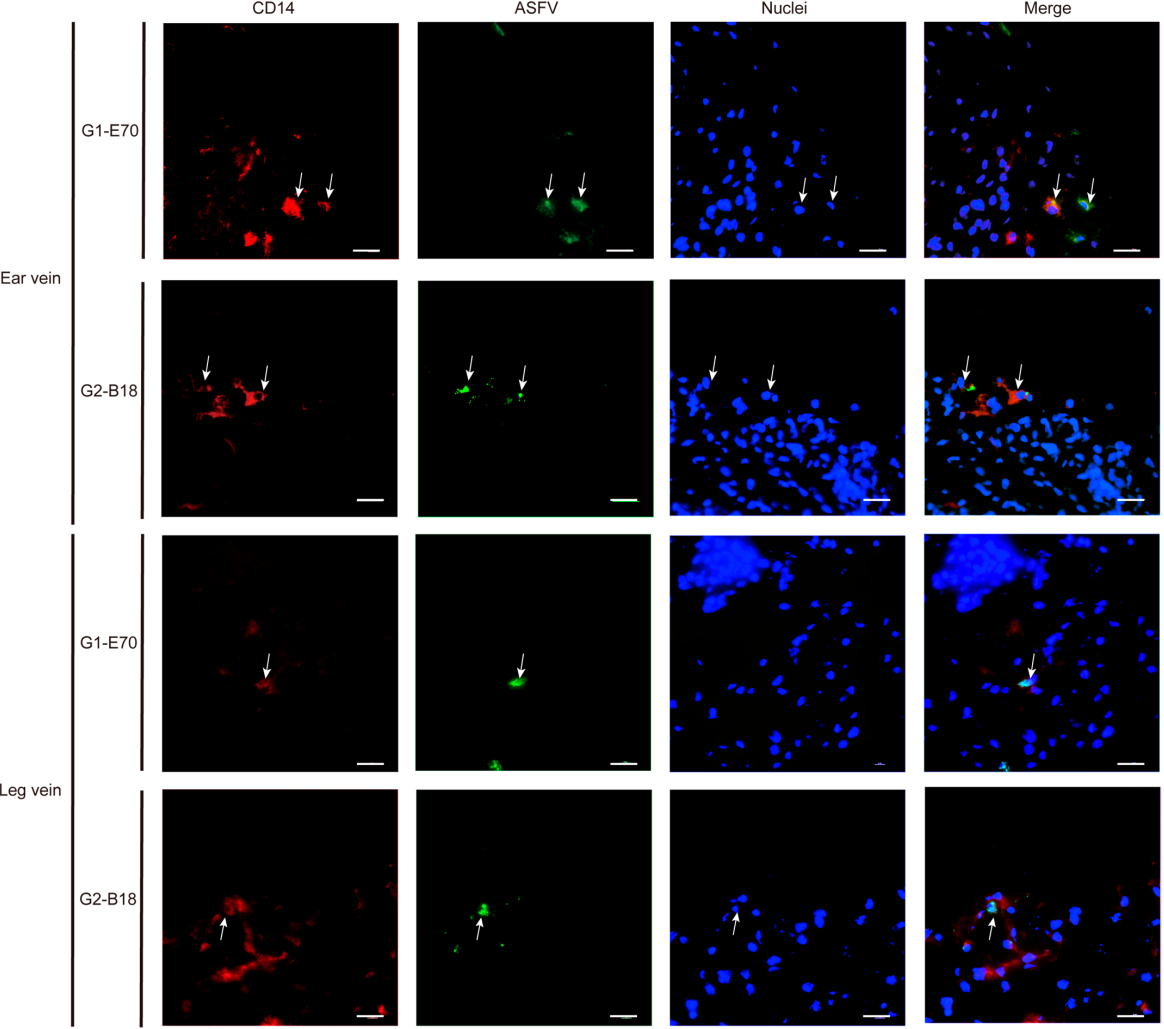
Expression of CD14 in ASFV-infected cells in ear and leg vein explants. Sections of the vein explants were stained with a double IF stain to identify the expression of CD14 (red) and ASFV (green) after being inoculated with G1-E70 or G2-B18. Blue represents cell nucleus staining. ASFV^+^CD14^+^ cells are indicated by white arrows. Scale bar: 50 μm.

**Figure 11:**
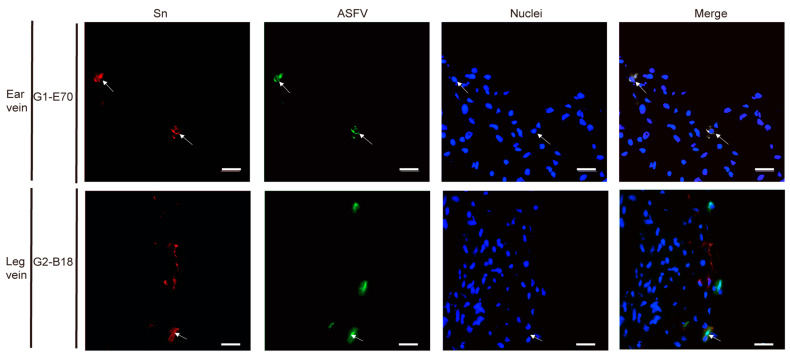
Identification of Sialoadhesin (Sn) in viral ASFV-infected cells in ear and leg vein explants. Sections of the vein explants were stained with a double IF stain to identify the expression of Sn (red) and ASFV (green) after being inoculated with G1-E70 or G2-B18. Blue represents cell nucleus staining. ASFV^+^Sn^+^ cells are indicated by white arrows. Scale bar: 50 μm.

**Figure 12:**
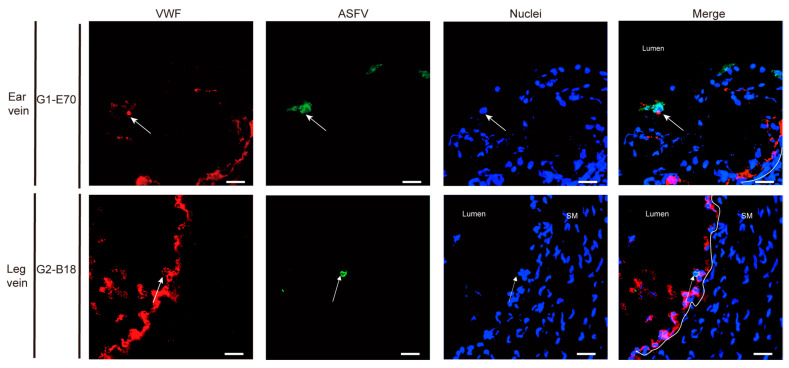
Expression of VWF in viral ASFV-infected cells in ear and leg vein explant. Sections of the ear vein explant were stained with a double IF stain to identify the expression of VWF (red) and ASFV (green) after being inoculated with G1-E70 or G2-B18. SM: Smooth muscle cells. Blue represents cell nucleus staining. ASFV^+^VWF^+^ cells are indicated by white arrows. Scale bar: 50 μm.

In the ear vein explant, most of the ASFV-infected cells were CD163^+^: 93.04% of the G1-E70 infected cells and 100% of the G2-B18 infected cells. Additional staining for cell surface markers indicated that 84.85% of the G1-E70 infected cells and 50% of the G2-B18 infected cells were vimentin^+^ (Figure 9); 51.91% of the G1-E70 infected cells and 75% of the G2-B18 infected cells were CD14^+^ (Figure 10); a few viral antigen-positive cells were Sn^+^ (8.89%) and VWF^+^ (2.56%) in the G1-E70 inoculated group (Figure 11 and Figure 12).

In the leg vein explant, also most of the ASFV-infected cells were CD163^+^: 75.16% of the G1-E70 infected cells and 96.97% of the G2-B18 infected cells. After the complementary cell surface markers staining, 49.68% of the G1-E70 infected cells and 73.11% of the G2-B18 infected cells were vimentin^+^ (Figure 9), and 59.63% of the G1-E70 infected cells and 26% of the G2-B18 infected cells were CD14^+^ (Figure 10). In addition, some infected cells were Sn^+^ (3.03% and 22.42%, respectively) and VWF^+^ (0.90% and 5.56%, respectively) in the G1-E70 and the G2-B18 inoculated explants (Figure 11 and Figure 12). In conclusion, ASFV mainly infects CD163^+^ cells that are sialoadhesin negative but CD14/vimentin positive. Endothelial cells can be exceptionally infected.

## 4. Discussion

Some pathogenic microorganisms that have a tropism for certain monocytes/macrophages and circulate in the blood may cause directly or indirectly severe vascular damage. Macrophages are the major immune cells distributed in the vascular area and exhibit different functions, including phagocytosis of microorganisms, antigen presentation, and the production of cytokines and chemokines [39]. Blood vessel pathologies were reported during infections with ASFV and PRRSV [11,40], but insights into the pathogenesis are still lacking. Previous research has indicated different levels of susceptibility among perivenous macrophages to PRRSV [34]. Up till now, the contribution of infected perivenous macrophages to the pathogenesis of hemorrhages during ASFV infections has not been studied. In our research, we examined this part of the pathogenesis of African swine fever (ASF) for the first time, using a vein explant model with ear and leg veins.

We initially assessed the viability of culturing vein explants. Most cells of the different components (endothelial cell layer, smooth muscle cell layer, and connective tissue) remained sufficiently viable for 72 h of in vitro cultivation. The infection of ASFV did not significantly affect the cellular viability in different tissue regions.

The pathogenesis of ASF mainly depends on the susceptibility of cells from the monocyte/macrophage lineage and is highly dependent on their origin, maturation stage, and activation status [12,41]. Multiple receptors and mediators are involved in facilitating the entry of ASFV into susceptible cells. CD163, a surface marker predominantly found on tissue macrophages [42], has been suggested to serve as a mediator for ASFV [21], suggesting that CD163^+^ macrophages exhibit a higher susceptibility to ASFV compared to other cells. In our study, we observed a widespread distribution of CD163^+^ cells in the connective tissue surrounding vein explants, with the majority of cells being present within a radius of 348 µm and decreasing further away from the vein. In the context of a human study, CD163^+^ cells were likewise detected in the perivascular regions of healthy skin tissue [43]. Circulating monocytes will infiltrate perivascularly and differentiate into macrophages [39], which may explain the origin of CD163^+^ cells observed in between the endothelial cells and smooth muscle cells. Previous research reported that ASFV infection may result in a decrease in the expression of CD163 [44]. In our study, ASFV infection did not alter the quantity of CD163^+^ cells.

The lesions and related clinical signs (e.g., cyanosis of the ears) of ASF are strain-dependent. Vascular lesions are often observed with highly virulent strains [12,45]. In cases of subacute African swine fever, clinical signs are less obvious, but severe vasculopathy is still present [12]. In this study, we aimed to investigate the susceptibility of perivenous macrophages to European pathogenic ASFV genotype 1-E70 and genotype 2-B18 in order to gain a more comprehensive understanding of their role in vasculopathy. The number of infected cells in both the ear and leg vein explants gradually increased over time, suggesting that the virus successfully replicated in vitro. In a previous study, ASFV was found to induce apoptosis in the cells it infects [46], and more importantly, apoptotic bodies were shown to facilitate virus spread [47]. Since perivenous macrophages are crucial for the maintenance of the microenvironment of veins [48], the susceptibility of perivenous macrophages to ASFV probably contributes to the vascular pathology (i.e., hemorrhage) related to ASFV infection. Inflammatory cytokines released from monocytes and macrophages might induce vascular leakage or increase vascular permeability [49]. Previous studies have identified elevated levels of cytokines, such as IL-1, IL-6, and TNF, in ASFV-infected tissues associated with hemorrhages [50,51]. Further research should be undertaken to investigate the biological significance of cytokines released from the ASFV-infected cells to the vessel pathology.

Our study revealed some differences in tissue tropism between genotype 1-E70 and genotype 2-B18. More infected cells were observed in the G1-E70 inoculated explants than in the G2-B18 inoculated explants. This pattern was the opposite of that in nasal explants. Indeed, in nasal explants, G2-B18 replicated better than G1-E70 [28]. This difference is most probably due to a difference in the number of non-macrophage targets. Indeed, epithelial cells became also infected in the nasal mucosa, and G2-B18 infected them at a higher level than G1-E70.

CD163-knockout pigs are susceptible to ASFV [22], indicating at least the existence of alternative mediators in the interaction between ASFV and susceptible cells [52,53]. This does not imply that the involvement of CD163 has to be ruled out. The existence of different receptors/entry mediators should be considered and may be the reason for the infection of non-macrophage targets.

Although the majority of cells infected by ASFV in vein explants were CD163^+^, some ASFV^+^CD163^−^ cells were also detected. As a result, the present study made efforts to discover more cells in vein explants that are susceptible to ASFV. A high percentage of ASFV^+^ cells (26–75%) were CD14^+^. Most of the CD14-positive cells were also CD163-positive in the vein explants (additional information added in Figure S2). CD14 is present in monocytes and tissue macrophages [54]. We believe that blood monocytes infiltrate perivascular areas and differentiate them into tissue-resident macrophages [55].

A very low percentage of ASFV^+^ cells were MHCII^+^: 3.92% of G1-E70 infected cells in the ear vein. MHCII is commonly found on the surface of cells involved in presenting antigens, and it is believed to have a significant impact on ASFV replication. [56]. However, in the present study, most infected cells were MHCII-negative.

Important was the observation of some ASFV^+^VWF^+^ cells in vein explants. This is consistent with earlier findings indicating the presence of viral antigen-positive cells within the endothelial cell layer [11,57,58,59]. It is difficult to understand why only a few endothelial cells became infected. If they were fully susceptible, we would have expected many more infected endothelial cells. It is very well possible that only a certain subpopulation of endothelial cells becomes infected or that virus becomes transmitted from infected macrophages to endothelial cells during their close contact during migration. This transmission may occur through filopodia and over microtubules, as earlier described by [60,61]. The susceptibility of cells to the virus may vary based on (i) cell origin, (ii) culture method, and (iii) virus virulence. Vallée et al. found that the highly virulent ASFV strain Malawi Lil 20/1 efficiently infected and replicated in primary porcine aortic endothelial cells (PAEC) and bush pig endothelial cells (BPEC), leading to apoptosis in the infected endothelial cells [62]. Infected macrophages, together with infected endothelial cells, may explain in part the hemorrhages induced by ASFV. Previous research has indicated that ASFV is capable of replicating fibroblasts and smooth muscle cells located within small vessels in the late stages of infection [14]. However, in our study, no ASFV-infected cells were observed in smooth muscle cells of veins. This vein model has demonstrated its functionality to enhance the understanding of the mechanisms of ASFV and other macrophage-tropic viruses in their development of vasculopathy in domestic pigs.

## 5. Conclusions

In summary, using an established vein explant model, we have examined the replication characteristics of ASFV genotype I E70 (G1-E70) and ASFV genotype II Belgium 2018/1 (G2-B18) in the environment of small veins. G1-E70 replicated more efficiently than G2-B18 in the vein explants. Mainly ASFV^+^CD163^+^ cells were observed in the explants. Further analysis of cells infected with ASFV showed the existence of vimentin^+^, MHCII^+^, CD14^+^, and VWF^+^ cells, indicating the diverse and complex cellular response to ASFV infection. These results make a significant contribution to enhancing our knowledge of the mechanisms underlying ASFV-induced hemorrhage.

## Figures and Tables

**Table 1:** Combination of antibodies used for immunofluorescence staining.

Primary Antibodies 1	Primary Antibodies 2						
	Name	Host	Target Cells	Clone	Isotype	Dilution	Company
Anti-VP72, IgG2a	Anti-CD163 mAb	mouse	macrophages	2A10/11	IgG1	1:200	Bio-rad
	Anti-Sialoadhesin mAb	mouse	macrophages	41D3	IgG1	1:50	[37]
	Anti-vimentin mAb	mouse	mesenchymal cells	V9	IgG1	1:50	Bio-rad
Anti-VP72, IgG1	Anti-CD14 mAb	mouse	monocytes	MIL2	IgG2b	1:100	[38]
	Anti-MHCII mAb	mouse	APCs	MSA3	IgG2a	1:200	Kingfisher Biotech
	Anti-smooth muscle actin mAb	mouse	smooth muscle cells	1A4	IgG2a	1:50	Dako
	Anti-von willebrand factor pAb	rabbit	endothelial cells		IgG	1:50	Dako

mAb: monoclonal antibody, pAb: polyclonal antibodies.

## Data Availability

The authors can provide the data supporting the findings of this study upon request.

## Supplementary Materials

The following supporting information can be downloaded at: https://www.mdpi.com/article/10.3390/v16101602/s1, Figure S1: Positive control for double immunofluorescence staining. Figure S2: Double immunofluorescence staining against CD163 (green) and CD14 (red) in vein explant. Table S1: Immunofluorescence staining for the selected cell markers was performed on lung and vein tissue sections.
