# Supplementary material for: Replication Characteristics of African Swine Fever Virus (ASFV) Genotype I E70 and ASFV Genotype II Belgium 2018/1 in Perivenous Macrophages Using Established Vein Explant Model
Source: Viruses. 2024 Oct 12;16(10):1602. doi: 10.3390/v16101602 (PMC11512260; doi:10.3390/v16101602)
Supplement: Supplementary file 1 [file viruses-16-01602-s001.zip › viruses-3240856-supplementary.pdf]

Supplementary Materials

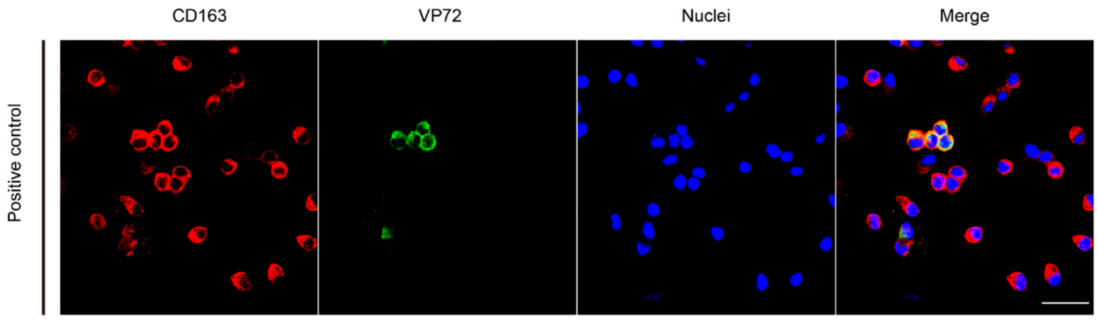

Figure S1. Positive control for double immunofluorescence staining. PAMs inoculated with ASFV were used as a positive control for the CD163/VP72(18BG3) double immunofluorescence staining. CD163 (red), VP72 (green), and nuclei (blue). Scale bar: 50 µm.

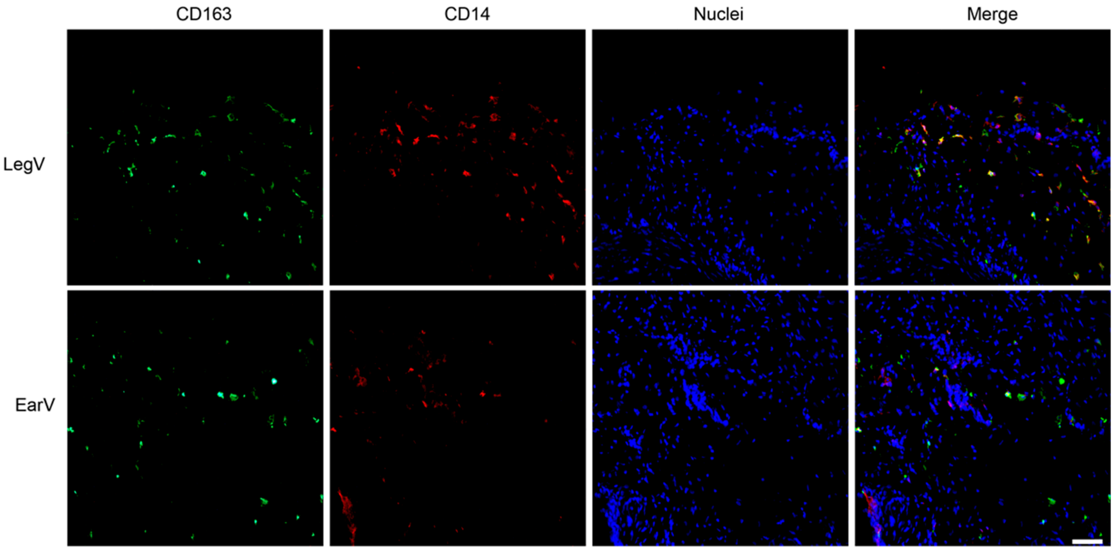

Figure S2. Double immunofluorescence staining against CD163 (green) and CD14 (red) in vein explant. Most CD14 positive cells were shown CD163 positive (76% in leg vein and 72% in ear vein). Scale bar: 50 µm.

Table S1. Immunofluorescence staining for the selected cell markers was performed on lung and vein tissue sections.

		Reactivity	
Markers	Cell types	Lungs	Veins
CD163	MΦ, Mo	+++++	+++
CD14	MΦ, Mo	+	++
Sn	MΦ	+++++	++
Vimentin	MSc, Fi, MΦ	+++++	+++++
MHCII	APC	+++	+
VWF	En	-	+++++
SMC	SMC	-	+++

Mo: Monocyte, MΦ: Macrophage, MSc: Mesenchymal cell, Fi: Fibroblast, APC: Antigen-presenting cell, En: Endothelial cell, SMC: Smooth muscle cells, Sn: Sialoadhesin, VWF: von Willebrand Factor. Reactivity signal is presented in plus and minus symbols.
